# A112 MEASURING COLORECTAL POLYP SIZE USING A NOVEL VIRTUAL SCALE FUNCTION DURING ENDOSCOPIES: A CLINICAL PILOT STUDY

**DOI:** 10.1093/jcag/gwac036.112

**Published:** 2023-03-07

**Authors:** R Djinbachian, M Zarandi-Nowroozi, M Taghiakbari, D von Renteln

**Affiliations:** CHUM, Montreal, Canada

## Abstract

**Background:**

Although accurate polyp size measurement is essential for adequately managing patients, we still rely on the endoscopists' subjective visual estimation of polyp size in routine practice.

**Purpose:**

We therefore conducted a clinical pilot study to evaluate relative accuracy of measuring colorectal polyp size during live colonoscopies using a novel virtual scale endoscope (VSE) that superimposes a virtual linear or circular ruler onto objects in the endoscopist's field of view.

**Method:**

We conducted a pilot phase 1 study to evaluate the relative accuracy of VSE for polyp size measurement. We measured polyp size from fresh polypectomy specimens before formalin fixation using a digital vernier caliper. These reference measurements were compared to visual size estimations and VSE measurements obtained during live colonoscopies. The primary outcome was the relative accuracy of visual size estimation versus size estimation using VSE. The secondary outcome was the percentage of measurements that fall within 25% of true polyp size.

**Result(s):**

43 patients (mean age: 63.3 years; 44.2% male) undergoing screening, surveillance, or diagnostic colonoscopies were included in the pilot study. Colonoscopies were performed by two endoscopists, including one trainee and one staff gastroenterologist. Visual, VSE, and reference size measurements was performed for 17 polyps. VSE during live colonoscopies had a higher relative accuracy of polyp size measurements when compared with visual polyp size estimation 84.3% (95% confidence interval (CI) 77.22% to 91.41%) vs 69.3% (95%CI 61.13% to 77.39%) (p=0.005). The percentage of size measurements that were within 25% of sizes as measured by caliper were 41.2% for visual polyp size estimation and 88.2% for VSE (p=0.02).

**Conclusion(s):**

We found that a novel endoscope equipped with a virtual scale allowed for significantly higher accuracy in polyp size measurement. A prospective randomized controlled trial is currently underway to validate these results.

**Please acknowledge all funding agencies by checking the applicable boxes below:**

Other

**Please indicate your source of funding below:**

Fujifilm

**Disclosure of Interest:**

R. Djinbachian: None Declared, M. Zarandi-Nowroozi: None Declared, M. Taghiakbari: None Declared, D. von Renteln Grant / Research support from: Daniel von Renteln is supported by a “Fonds de Recherche du Québec Santé” (FRQS) career development award and has received research funding from ERBE, Ventage, Pendopharm, Fujifilm, and Pentax., Consultant of: Boston Scientific and Pendopharm,

